# Unlocking the Potential of Animal Hair Shafts for Genomic Studies: A Comprehensive Evaluation of DNA Quality

**DOI:** 10.3390/biology14040353

**Published:** 2025-03-28

**Authors:** Yongheng Zhou, Qi Zhang, Peng Gao, Shuhui Yang, Yanchun Xu

**Affiliations:** 1 College of Wildlife and Protected Area, Northeast Forestry University, Harbin 150040, China; zhouyongheng0202@163.com (Y.Z.); 15735171383@163.com (Q.Z.); 18747443626@163.com (P.G.); yangshuhui@nefu.edu.cn (S.Y.); 2 National Forestry and Grassland Administration Research Centre of Engineering Technology for Wildlife Conservation and Utilization, Harbin 150040, China; 3 BGI Life Science Joint Research Centre, Northeast Forestry University, Harbin 150040, China

**Keywords:** hair shaft, non-invasive, genomic DNA, genetics and evolution

## Abstract

Hair is a prominent physiological feature of many animals that can be easily and non-invasively sampled. However, most previous studies have focused on human hair, and animal hair has not been widely used as a DNA source in genetic and evolutionary studies. Analysing the DNA quality of animal hair is more challenging than that of human hair because it varies across different species and is influenced by different storage conditions. To address this gap in our knowledge, this study systematically evaluated the DNA quality of various animal hair samples. Our findings show that lightly medullated hairs, which are typical in carnivores, yield greater amounts of DNA and have better sequencing results than highly medullated hairs, which are typical in ungulate herbivores. Furthermore, the roots and middle hair were more productive than the upper hair parts. Tanning and storage time impair DNA yield and sequencing quality and increase nucleotide damage. Moreover, for certain historical specimens and field samples, genomic DNA was more successfully obtained from hair samples than from skin samples. This study provides data and theoretical support for improving the use of animal hair in genetic research.

## Simple Summary

Despite being one of the most accessible and non-invasive biological materials, animal hair genomic DNA is not widely used as a primary source of genetic material. This underutilisation represents a waste of resources. Research to elucidate the quality of DNA obtained from animal hair samples is limited, significantly restricting its wider application in scientific research. To address this gap in our knowledge, we have evaluated the quality of DNA obtained from various animal hair samples. The results show that animal hair has an abundance of DNA, predominantly nuclear DNA. Genomic DNA in hair usually undergoes varying degrees of degradation, the extent of which is influenced by factors such as storage time, environmental conditions, and sample type. Furthermore, our study found that for some historical specimens and field samples, hair is a better option for obtaining genomic DNA. This study provides a deeper understanding of animal hair as a genetic resource and promotes its application in wildlife genetics.

## 1. Introduction

Hair, a common biological feature of animals, is widely distributed across the body surface, and its primary functions include protection and body temperature regulation. It can also be easily accessed and sampled non-invasively. Hair contains components such as hormones, proteins, and DNA, making it valuable in various fields, including animal nutrition, species identification, and environmental pollution monitoring [1,2,3]. However, genetic and evolutionary studies on DNA extracted from hair, particularly nuclear DNA (nuDNA), are limited. This is primarily due to the unsatisfactory results of previous genotyping studies (e.g., STRs, SNPs) [4,5,6], which have hindered its broader application in genetic research.

Hair contains DNA, despite its absence of “living” cells. This was evidenced by Szabo et al. [7], who used in situ DNA labeling techniques and observed irregular blue fluorescence spots in hair samples, which indicated that nuclear genetic material was bound to the DNA-specific dye Hoechst 33258. Subsequent studies revealed that freshly shed hair contains approximately 10 ng of total DNA; however, this significantly declines over time to <1 ng after several months [8]. Despite the presence of DNA in hair, its application remains limited. While mitochondrial DNA (mtDNA) genotyping from hair has been highly successful and produced results consistent with those from muscle tissue DNA [8,9,10], nuDNA genotyping often presents challenges, such as the presence of artefacts, missing signals, or erroneous peak patterns [11,12]. This may be because nuclear DNA in hair degrades during hair formation, whereas mtDNA within the mitochondria remains relatively intact, protected by the mitochondrial structure [13,14].

With the advancement of next-generation sequencing (NGS) technologies, it has become feasible to use fragmented DNA. Parson et al. [5] successfully obtained complete mtDNA sequences from a single shed hair, and their results were consistent with genomic data obtained from high-quality DNA samples using Sanger sequencing. Subsequent studies have confirmed that hair contains nuDNA, with quantities significantly greater than mtDNA. However, the average fragment length of hair nuDNA is 50–80 bp, considerably shorter than that of mtDNA [5,14,15,16]. This characteristic is one of the primary reasons that PCR amplification of hair nuDNA fails, as a PCR typically requires template DNA fragments of at least 200 bp.

Current genomic sequencing studies on hair have predominantly focused on human hair [5,15,16,17,18,19], with research on wild animal hair remaining scarce. Compared with human hair, animal hair exhibits greater diversity in types and is often subjected to more extreme preservation conditions [20]. Animal hairs can be used as valuable non-invasive samples in field surveys and historical specimen research because of their greater availability compared with other tissue types. However, there is limited understanding of the DNA quality in animal hair, and a lack of systematic evaluation has hindered its broader use in DNA research. Consequently, using hair as a DNA source in field and historical studies has not yet been widely accepted. Therefore, investigating the quality characteristics of animal hair DNA, particularly in relation to environmental factors, preservation conditions, and species differences, holds significant scientific and practical value.

To address the gaps in our knowledge, this study aimed to evaluate the genomic DNA quality of animal hair with rootless under various environmental conditions, including storage environment, tanning processes, preservation duration, and field conditions. We systematically assessed the DNA quality of animal hair using indicators such as the mapping rate (*R*_m_), average read length (*A*_L_), and multiple mapping rate (*R*_m0_). The results will enhance our understanding of animal hair genomic DNA quality and provide data-driven insights and theoretical support for its broader application in genetics, evolutionary research, and wildlife conservation.

## 2. Materials and Methods

### 2.1. Sample Selection

In total, 15 skin samples and 43 keratinized samples were collected from the remains of wild or captive animals. The sequencing data for these samples were sourced from the National Centre for Biotechnology Information (NCBI) under project IDs PRJNA1190142 and PRJNA1103004. All hair samples were processed for sequencing after removing the root (cuts were made a minimum of 0.5 cm from the root end). Further details are presented in Table S1.

### 2.2. Quality of Sequencing Data from Hair Shaft DNA

The sequencing quality of DNA from hair shafts was evaluated for different types, sections of hair, and tanning degrees using the mapping rate (*R*_m_), ratio of multi-mapped reads to aligned reads (*R*_m0_), and average size of reads (*A*_L_).

#### 2.2.1. Different Types of Hair Shaft

We chose the hairs of three Siberian roe deer (H1, H2, and H3), sika deer (H4), red deer (H5), and Eurasian moose (H6) as representatives of coarse and highly medullated hair, and the hairs of four Amur tigers (H7, H8, H9, and H10), two wolves (H11 and H12), an Arctic fox (H13), and a leopard cat (H14) as representatives of fine and lightly medullated hair (Figure S1) [21]. Additionally, the guard hair (H15) and underfur (H16) from an Arctic fox was selected for comparison between the two types of hairs.

#### 2.2.2. Different Sections of Hair Shaft

To evaluate the sequencing quality of DNA from different parts of the hair, 10 rootless guard hairs were chosen from the Arctic fox (H17) and sika deer (H18) and cut into three equal sections: basal (A), middle (B), and distal (C).

#### 2.2.3. Different Tanning Degree

**Figure 1:**
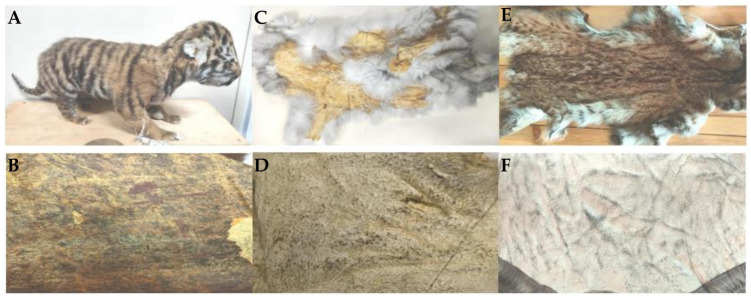
Skin samples with three tanning degrees. Level-1: non-tanned (**A**,**B**); Level-2: moderately tanned (**C**,**D**); Level-3: fully tanned (**E**,**F**). Upper images: condition of the hair (pelts); lower images: condition of the skins.

To evaluate the influence of tanning on sequencing quality, hair (including both guard and underside hair) was sampled from 13 skins that were sorted into three tanning degrees. Level-1 were untanned and naturally dried skins including two Amur tigers (H19, H20), a leopard cat (H21), a wolf (H22), a Siberian roe deer (H23), a red deer (H24), and an Eurasian moose (H25) (Figure 1A,B); Level-2 were moderately tanned including a yellow-throated marten (H26), a mountain hare (H27), a red fox (H28), and a wolf (H29) (Figure 1C,D); and Level-3 were fully tanned including two leopard cats (H30, H31) (Figure 1E,F). Additionally, we used the DNA from skin preserved at −20 °C (S1~S13) as a control for each species.

#### 2.2.4. Hair Shaft on Decaying Carcass

To evaluate the sequencing quality of hairs on decaying carcasses in the wild, we chose carcasses of a wolf (H32, S14) and a Siberian roe deer (H33, S15) that were suspected to have been dead for over six months and subsequently stored at −20 °C for at least one year.

#### 2.2.5. Hair Shaft in Faeces

To evaluate the quality of the sequencing data from hair digested in the digestive tract, we chose five hair samples (H34–H38) from feces, compared with five naturally shed hair samples (H39–H43) to perform and assess resequencing. Hairs from feces were repeatedly washed 8–10 times with 0.1% enzyme-containing laundry detergent solution to remove fecal residues.

### 2.3. Analysis of DNA Damage

DamageProfiler v1.1 [22] was used to model DNA damage in hair samples that had been stored for 45 to 60 years in a room environment without special control of temperature and humidity. The main indices for comparison included the number of significantly damaged bases at the 3′ end (*N*_db_, the consecutive number of bases from the end of the sequence with damage rates higher than the average damage rate), the highest damage rate of bases at the 3′ end of the sequence (*R*_dm_) and the coefficient of variation (CV) among hair samples.

### 2.4. Data Analysis and Statistics

We applied the same data processing pipeline to both the hair and skin samples. First, raw sequencing data were trimmed using Trimmomatic 0.39 [23] to remove low-quality bases and adapter sequences. The trimmed data were then assessed for quality using FastQC v0.11.9 [24] to ensure that all adapter sequences were effectively removed. Based on our research experience, it is characterized by significant fragmentation of DNA from animal hair shaft, and the BWA aln 0.7.17-r1188 [25] was used to align the sequencing reads to the reference genome and generate .bam files. Finally, relevant metric statistical analyses were performed using tools such as BAMDST 1.0.9 [26], GATK 4.2.1.0 [27], and SAMtools 1.6 [28].

All indices were calculated using scientific notation. The Shapiro–Wilk test was used to test data normality. The nonparametric Wilcoxon signed-rank test was used for the statistical analysis of datasets that were significantly nonnormally distributed, whereas the parametric *t*-test was used to analyse normally distributed datasets. The alpha threshold was set at 0.05 for all analyses. R 4.2.3 and OriginPro 2021 were used for all calculations, classifications, and graphical visualisation of the results.

## 3. Results

### 3.1. Different Types

In the comparison between lightly and highly medullated hairs, the *R*_m_ for lightly medullated hairs was between 71.13% and 90.88%, with an average of 83.13% ± 7.34%. The *R*_m0_ varied from 14.48% to 66.23% and averaged 34.35% ± 15.13%. The *A*_L_ ranged between 33.7 and 71.9 bp with a mean of 50.3 ± 12.6 bp. In highly medullated hairs, the *R*_m_ was between 16.45% and 79.59%, with an average of 44.05% ± 22.35%. The *R*_m0_ averaged 48.61% ± 12.80%, varying from 36.93% to 73.82%, and the *A*_L_ averaged 43.1 ± 7.1 bp and varied between 34.7 bp and 54.0 bp. Statistically, *R*_m_ was significantly greater in lightly medullated hair than in highly medullated hair (*t-*test: |t| = 4.675, *p* = 0.001), whereas *R*_m0_ and *A*_L_ were not significantly different (Wilcoxon test-*R*_m0_: *p* = 0.020; *A*_L_: |t| = 1.252, *p* = 0.234).

**Figure 2:**
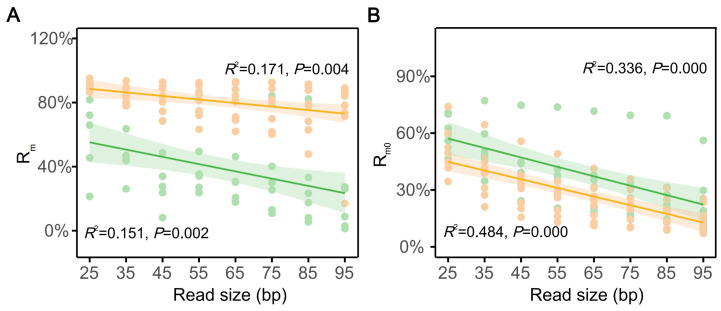
The relationship between *R*_m_ (**A**) and *R*_m0_ (**B**) and read size in two types of mammalian guard hairs. A serial size ranges were subsampled with a step length of 10-bp from the total cleaned reads of each sample, and each range was represented by the middle value shown as a dot. The yellow section represents lightly medullated hairs, whereas the green section represents highly medullated hairs. The shading ranges show the 95% confidence interval.

Further analysis revealed that R_m0_ and R_m_ were sensitive to read size, showing strong negative correlations with both lightly (*R*_m0_: *R*^2^ = 0.484, *p* = 0.000; *R*_m_: *R*^2^ = 0.171, *p* = 0.004) and highly medullated hair shafts (*R*_m0_: *R*^2^ = 0.336, *p* = 0.000; *R*_m_: *R*^2^ = 0.151, *p* = 0.002) (Figure 2). The trends were similar between lightly and highly medullated hairs; however, lightly medullated hairs performed better in Rm with increasing read sizes. For example, the *R*_m_ could be as high as 72.15% ± 23.55% (ranging from 16.98% to 89.03%) when *A*_L_ exceeded 90 bp, whereas it remained at 25.12% ± 31.06% (1.33% to 84.23%) in highly medullated hairs.

Additionally, we compared the DNA quality between the guard hair and underfur. The proportions of endogenous DNA (*R*_m_) were similar: 93.37% in the guard hair and 92.24% in the underfur hair. The average size of the reads (*A*_L_) of the guard hairs was 47.3 bp, which was slightly lower than that of the underfur (54.6 bp). This suggests that the quality of the DNA of the underfur hair was slightly higher than that of the guard hair.

### 3.2. Different Sections

**Figure 3:**
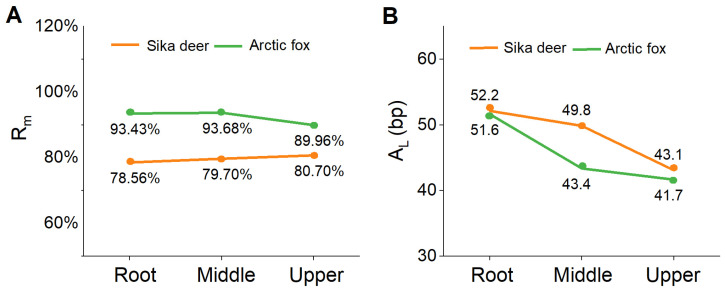
Variation in sequencing quality along the shaft of guard hairs of an Arctic fox and a sika deer. (**A**) mapping rate *R*_m_; (**B**) average read size *A*_L_.

We evaluated variations in the sequencing quality of the root, middle, and upper sections of guard hairs using Arctic fox and sika deer as representatives of lightly and highly medullated hairs. The results demonstrated *R*_m_ was stable across the three sections (Figure 3A), whereas *A*_L_ decreased slightly from the root to the upper part, with a difference of approximately 10 bp (Figure 3B). These findings suggest that the genomic DNA content is even along the hair shaft, whereas the degree of fragmentation increases towards the tip.

### 3.3. Effect of Degree of Tanning

**Figure 4:**
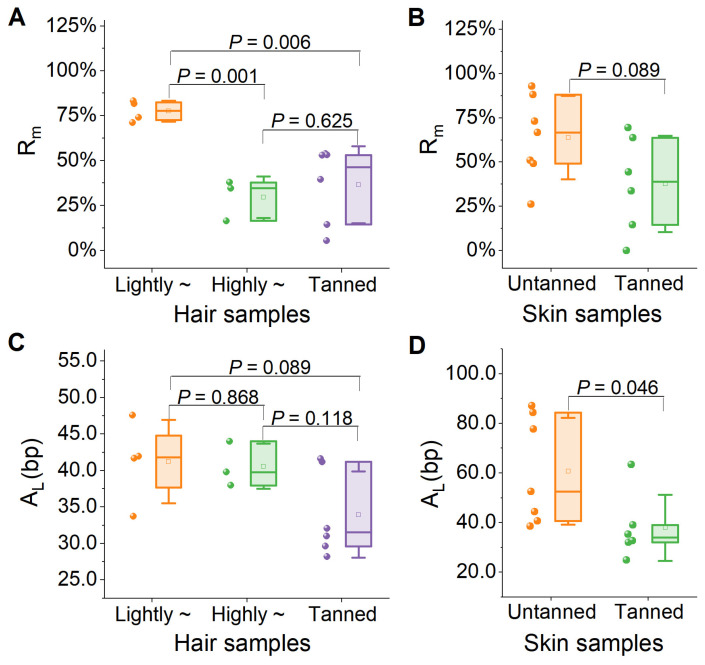
Effects of tanning on the genomic sequencing quality of hair shafts and skin. (**A**) Mapping rate of reads (*R*_m_) for hair samples; (**B**) mapping rate of reads (*R*_m_) for skin samples; (**C**) average read size (*A*_L_) for hair samples; (**D**) average read size (*A*_L_) for skin samples. Lightly ~ represents untanned, low-medullated hair samples, whereas highly ~ denotes untanned, high-medullated hair samples.

The historical specimens were classified into three categories based on the tanning degree: non-tanned, slightly tanned, and deeply tanned skins (pelts). Rm exhibited a dramatic declining trend as the tanning deepened. The average Rm was 57.00% ± 26.76% for hair from non-tanned skins (77.52% ± 5.85% for lightly medullated hairs and 29.64% ± 11.54% for highly medullated hairs), 49.90% ± 6.94% for hair from slightly tanned skins, and 9.94% ± 6.29% for hair from deeply tanned skins. The proportion of endogenous DNA in untanned and low-medullated hair was significantly higher than that in high-medullated hair (*t*-test: |t| = 7.300, *p* = 0.001) and tanned hair (*t*-test: |t| = 3.650, *p* = 0.006). However, no significant difference was observed between high-medullated hair and tanned hair (*t*-test: |t| = 0.511, *p* = 0.625) (Figure 4A). A similar trend was observed for the skin from which the hair was sampled. The Rm was 63.86% ± 23.50% for non-tanned skins (68.45% ± 30.35% for lightly medullated hairs and 57.76% ± 13.30% for highly medullated hairs), 52.82% ± 16.68% for hair from slightly tanned skins and further dropped down to 7.34% ± 10.27% in hair from deeply tanned skins, even below the level obtained from the hair of the deeply tanned pelts, although the difference between tanned and untanned pelts was not statistically significant (*t*-test: |t| = 1.866, *p* = 0.089) (Figure 4B). These results suggest that the rate of *R*_m_ decline is slower in the hair shafts than in the skin itself.

Regarding DNA fragmentation, the overall *A*_L_ in hair shafts obtained from non-tanned skins (pelts) was 41.0 ± 4.4 bp, specifically 41.2 ± 5.7 bp for lightly medullated hairs and 40.6 ± 3.1 bp for highly medullated hairs, respectively. The *A*_L_ value declined to 35.2 ± 7.2 bp in hairs from slightly tanned skins and further to 31.6 ± 0.8 bp in hairs from deeply tanned skins. No significant differences among untanned high-medullated hair, untanned low-medullated hair, and tanned hair were observed (*t*-test: |t| = 0.175, *P*_Lvs_._H_ = 0.868; |t| = 1.934, *P*_Lvs_._T_ = 0.089; |t| = 1.784, *P*_Hvs_._T_ = 0.118) (Figure 4C). In the skin samples, the overall *A*_L_ was 60.8 ±21.5 bp for non-tanned skins (66.2 ± 23.1 bp for skins with lightly medullated hairs and 53.65 ± 21.2 bp for skins with highly medullated hairs). This index declined to 42.5 ± 14.3 bp for slightly tanned skins and 28.8 ± 5.6 bp for deeply tanned skins. Notably, the *A*_L_ of hair shafts was 2.8 ± 4.8 bp longer than that of skins in the case of deeply tanned pelts. The trend in the decline in *A*_L_ was similar between the hair and skin (Figure 4B). Untanned skins exhibited significantly higher values compared to tanned skins (*t*-test: |t| = 2.252, *p* = 0.046) (Figure 4D). These results suggest that the integrity of DNA is affected by tanning and that DNA is better preserved in the hair shaft than in the skin during tanning.

### 3.4. Hair Samples from Decaying Carcass and Digestive Tract

To evaluate the sequencing quality of hair shafts from decaying carcasses, a wolf and roe deer that had been deceased for over six months in their natural environment were used as sources of lightly and highly medullated hair. We showed that *R*_m_ was 88.38% for wolf hair and for 60.47% roe deer hair. The index was 37.71% for wolf skin and 38.70% for roe deer skin. The *A*_L_ was 46.0 bp for wolf hairs and 34.7 bp for roe deer hairs, whereas skin samples had higher *A*_L_ values (91.0 bp for wolf and 63.1 bp for roe deer). This suggests that the sequence integrity (*A*_L_) of hair DNA was slightly lower than that of skin samples and that the proportion of endogenous DNA (*R*_m_) in hair samples was significantly higher.

A similar evaluation of hairs recovered from carnivore faeces showed that *R*_m_ ranged from 82.31% to 85.09% and averaged 84.16% ± 1.13%. This level was similar to that of naturally shed hairs that ranged from 78.98% to 84.10% with a mean of 82.17% ± 1.91% (*t*-test: |t| = 2.000, *p* = 0.081). Similarly, *A*_L_ did not show any difference either (Wilcoxon test-*p* = 0.548), where it averaged 74.2 ± 2.5 bp in faecal hairs and 73.8 ± 2.7 bp in naturally shed hairs. This indicates that digestion in the digestive tract of carnivores does not alter DNA quality in the hair shaft.

### 3.5. DNA Damage

In this study, hair samples were classified into six types: fresh, low-medullated hair, fresh high-medullated hair, historical low-medullated hair, historical high-medullated hair, tanned hair, and hair from faeces. When comparing DNA damage across these categories, tanned hair exhibited the highest damage rate at 3.23% ± 0.95%. The damage rates for historical high-medullated hair, fresh high-medullated hair, and historical low-medullated hair were similar, at 2.96% ± 1.18%, 2.71% ± 1.69%, and 2.48% ± 0.43%, respectively. Fresh low-medullated hair had a lower damage rate of 1.87% ± 0.74%, whereas hair collected from faeces exhibited the least DNA damage, with a rate of only 1.49% ± 0.30%.

**Figure 5:**
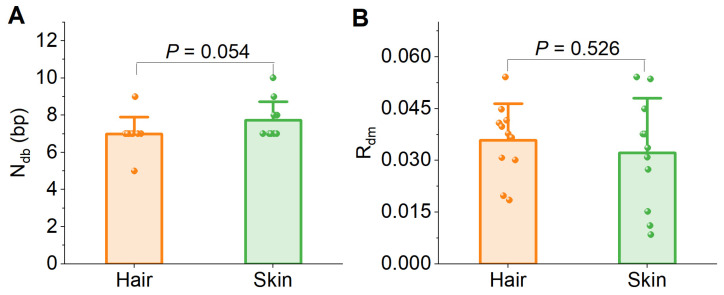
DNA damage in long-stored hair and skin DNA. (**A**) Comparison of the number of damaged bases (*N*_db_); (**B**) Comparison of the maximum damage rate (*R*_dm_).

Next, we evaluated the degree of DNA damage in long-stored samples by comparing hair shaft and skin samples stored for 45–60 years. We show that the number of significantly damaged bases (*N*_db_) at the ends averaged 7.0 ± 0.8 bp in the hair DNA. This number was similar to the 7.3 ± 1.0 bp in skin DNA, with no statistically significant (paired Wilcoxon test: *p* = 0.054). The CV of *N*_db_ were similar for hair (0.128) and skin DNA (0.130) (Figure 5A). The maximum rate of damage (*R*_dm_) averaged 0.036 ± 0.011 in hair DNA and 0.004 ± 0.019 in skin DNA, the difference was not significant (paired *t*-test: |t| = 0.657, *p* = 0.526). However, the CV of *R*_dm_ in hair DNA (0.294) was much lower than that in skin DNA (0.494). The progress of DNA damage during preservation was similar between hair shafts and skin, but the damage rate was more stable in the hair shaft (Figure 5B). These results suggest that hair is slightly superior to skin in terms of the preservation of DNA.

## 4. Discussion

Keratinized materials have different chemical components, microstructures, and physicochemical properties [29,30,31]. Keratinolytic organisms such as bacteria, fungi, and insects, as well as chemical or physical factors such as UV radiation, tanning agents, and metal catalysts, can decompose keratins, leading to the loss of DNA [32,33,34]. The variation in natural DNA remnants during cellular keratinisation and the extent of such bio- and abio-degrading factors, together with their different resistances, may result in variations in the yield and quality of DNA. For example, a lightly medullated hair shaft (typical in carnivores) generally produces a higher *R*_m_ (Figure 2) and a longer *A*_L_ than does a highly medullated one typical of ungulate herbivores. Hairs were directly sampled from shortly stored untreated skin (Table S1). Thus, this difference was mainly attributed to the microstructure. Lightly medullated hairs often contain a tthe hick cuticle, cortex, and dense medulla compared to highly medullated hairs, and nuclear DNA is proposed to be present in the cuticle and cortex [4,12]. Therefore, it is reasonable to speculate that genomic sequencing of ungulate herbivores would consume a greater amount of hair samples than carnivores, especially for samples decaying in nature for a time when keratinolytic organisms are likely to destroy highly medullated hair shafts and result in less recovery of DNA.

Historical specimens provide crucial data for genetic and evolutionary studies. The results of this study indicate that low-medullated hair from historical specimens contained endogenous DNA in proportions similar to that of muscle tissue, with a fragmentation degree slightly less than that of muscle tissue (Figure 4). Furthermore, the data characteristics of the hair samples were relatively stable. In contrast, high-medullated hair has significantly lower DNA quality than the corresponding muscle tissue. Tanning significantly affected DNA recovery and sequencing quality (Figure 4). This reduction was even greater in highly medullated hairs than in lightly medullated hairs (Table S2), suggesting that thinner cuticles and cortices and more porous medullae are sensitive to tanning agents. The difficulty of using hair shafts on tanned specimens depends on the degree of tanning. Moreover, the upper part of the hair is directly exposed to UV and environmental chemicals, whereas the root part is embedded and well protected in the fur coat. It was not surprising that DNA recovery and sequencing quality tended to decline from the root to the tip (Figure 3). This is in agreement with previous experiments on DNA extraction and the genotyping of genetic markers [35,36,37]. However, these effects were not notable unless the exposure was sufficient.

In natural environments, wild animals may die unexpectedly due to various factors. The complex and diverse microbial environment in the wild accelerates the decomposition of animal carcasses, leading to significant DNA degradation, which negatively impacts DNA extraction from skin tissue. However, in this study, genomic DNA was successfully extracted from the hair samples of two severely decomposed carcasses, with endogenous DNA proportions of 88.38% (wolf) and 60.47% (roe deer). In contrast, there were bead aggregation issues when extracting the DNA from the muscle tissues of these animals, severely reducing DNA yield, with endogenous DNA proportions < 40%. These results suggest that hair samples exhibit better resistance to decomposition than muscle tissue. This may be because the dry environment of hair and its unique keratin structure help slow DNA degradation. Therefore, for carcasses in the advanced stages of decomposition, hair samples may provide a more reliable source of stable DNA.

Faeces are another important material for DNA retrieval in the wild. However, faeces that are not collected in a timely manner are rapidly decomposed by microorganisms [38], whereas the hair within the faeces remains intact. Our further assessment of genomic DNA quality in faecal hair revealed no significant differences compared with untreated hair samples (Table S3). This finding provides a promising new direction for DNA collection in field studies.

In this study, we classified common hair types into six categories (historical low- and high-medullated hair, fresh low- and high-medullated hair, tanned hair, and hair found in faeces). A common characteristic of these samples is the varying degrees of DNA fragmentation, closely resembling ancient DNA (aDNA) [20,39]. A key feature of aDNA is DNA damage, which involves cytosine deamination. Similarly, different levels of DNA damage were observed in these six hair types, especially in historical and tanned samples. Interestingly, hair from older faeces exhibited a lower DNA damage rate than fresh hair samples. This could be attributed to the long-term storage of faecal samples at −80 °C or the protective properties of residual material, which may have delayed the degradation process. This further highlights the potential value of faecal-coated hair samples in field studies.

Long-term storage also allows postmortem DNA modifications to occur in keratinized materials, similar to ancient bones [40]. From the hair samples stored for 45 to 60 years in a room environment without control of temperature and humidity, we detected 7.0 ± 0.8 bp end damage on average, marginally similar to skins (7.3 ± 1.0 bp) in the same environment (Figure 5A). However, the maximum damage rate (*R*_dm_) in hair DNA did not fluctuate as in skin DNA (Figure 5B), suggesting that the damage progress s in such keratinized material is more stable than in soft tissues. Nevertheless, end damage to DNA should be taken into consideration when old hair is used for genomic sequencing, even though they are not ancient archaeological samples.

## 5. Conclusions

This study systematically analzsed the DNA quality of 43 hair shaft samples, revealing the impact of multiple factors such as hair type, storage conditions, and tanning on DNA quality. Our findings show that low-medullated hairs, typical of carnivores, yield greater amounts of DNA and have better sequencing quality than high-medullated hairs, typical of ungulate herbivores. Additionally, the roots and middle parts of the hairs were more productive than the upper parts. Tanning and storage time impair DNA yield and sequencing quality and increase nucleotide damage. Importantly, exposure to the digestive tracts of predators did not influence usability and DNA in hair encased in faeces degrades more slowly than that in hair stored at room temperature. This study provides important data support and theoretical foundations for the application of DNA from hair shaft, particularly in genetic research involving historical and field samples.

## Data Availability

The data supporting the findings of this study have been deposited at the National Centre for Biotechnology Information (NCBI) under BioProject numbers PRJNA1190142 and PRJNA1103004.

## Supplementary Materials

The following supporting information can be downloaded at: https://www.mdpi.com/article/10.3390/biology14040353/s1, Figure S1: Scanning electron microscopy (SEM) images of different types of hair. (A) Red deer, (B) Siberian roe deer, representing coarse and highly medullated hair types; (C) Snow leopard, and (D) Leopard, representing fine and lightly medullated hair types. Scale bars: 80 μm; Table S1: Information of samples; Table S2: Information of sequencing data; Table S3: Information for different sample types.

## Abbreviations

The following abbreviations are used in this manuscript:

mtDNA	Mitochondrial DNA
nuDNA	Nuclear DNA
aDNA	Ancient DNA
NCBI	National Center for Biotechnology Information
NGS	Next-generation sequencing
