# Supplementary material for: Unlocking the Potential of Animal Hair Shafts for Genomic Studies: A Comprehensive Evaluation of DNA Quality
Source: Biology (Basel). 2025 Mar 28;14(4):353. doi: 10.3390/biology14040353 (PMC12024741; doi:10.3390/biology14040353)
Supplement: Supplementary file 1 [file biology-14-00353-s001.zip › biology-3518466-supplementary.pdf]

Table S1 Information of samples.

Sample ID	Species	Scientific Name	Sample Type	Preservation year up to the experiment	Source
Skin					
S1	Amur tiger	*Panthera tigris altaica*	Dried raw skin	~50	Wild
S2	Amur tiger	*Panthera tigris altaica*		~50	Zoo
S3	Leopard cat	*Prionailurus bengalensis*		~55	Wild
S4	Wolf	*Canis lupus*		~50	Wild
S5	Siberian roe deer	*Capreolus pygargus*		~45	Wild
S6	Red deer	*Cervus canadensis*		~55	Wild
S7	Eurasian moose	*Alces alces*		~45	Wild
S8	Yellow-throated marten	*Martes flavigula*	Tanned skin	~55	Wild
S9	Mountain hare	*Lepus timidus*		~60	Wild
S10	Red fox	*Vulpes vulpes*		~55	Wild
S11	Wolf	*Canis lupus*		~45	Wild
S12	Leopard cat	*Prionailurus bengalensis*		~55	Wild
S13	Leopard cat	*Prionailurus bengalensis*		~55	Wild
S14	Wolf	*Canis lupus*	Decaying skin	~1.5	Wild
S15	Siberian roe deer	*Capreolus pygargus*		~3	Wild
Rootles hair shaft					
H1	Siberian roe deer	*Capreolus pygargus*	Highly medullated hair	0.5	Wild
H2	Siberian roe deer	*Capreolus pygargus*	Highly medullated hair	~3	Wild
H3	Siberian roe deer	*Capreolus pygargus*	Highly medullated hair	~45	Wild
H4	Sika deer	*Cervus nippon*	Highly medullated hair	2.5	Farm
H5	Red deer	*Cervus canadensis*	Highly medullated hair	~55	Wild

H6	Eurasian moose	*Alces alces*	Highly medullated hair	~45	Wild
H7	Amur tiger	*Panthera tigris altaica*	Lightly medullated hair	2	Zoo
H8	Amur tiger	*Panthera tigris altaica*	Lightly medullated hair	~6	Zoo
H9	Amur tiger	*Panthera tigris altaica*	Lightly medullated hair	~50	Wild
H10	Amur tiger	*Panthera tigris altaica*	Lightly medullated hair	~50	Zoo
H11	Wolf	*Canis lupus*	Lightly medullated hair	~1.5	Wild
H12	Wolf	*Canis lupus*	Lightly medullated hair	~50	Wild
H13	Arctic fox	*Vulpes lagopus*	Lightly medullated hair	2.5	Farm
H14	Leopard cat	*Prionailurus bengalensis*	Lightly medullated hair	~55	Wild
H15	Arctic fox	*Vulpes lagopus*	Lightly medullated hair	2	Wild
H16	Arctic fox	*Vulpes lagopus*	Lightly medullated hair	2	Wild
H17	Arctic fox	*Vulpes lagopus*	Lightly medullated hair	2	Wild
H18	Sika deer	*Cervus nippon*	Highly medullated hair	2.5	Farm
H19	Amur tiger	*Panthera tigris altaica*	Lightly medullated hair	~50	Wild
H20	Amur tiger	*Panthera tigris altaica*	Lightly medullated hair	~50	Zoo
H21	Leopard cat	*Prionailurus bengalensis*	Lightly medullated hair	~55	Wild
H22	Wolf	*Canis lupus*	Lightly medullated hair	~50	Wild
H23	Siberian roe deer	*Capreolus pygargus*	Highly medullated hair	~45	Wild
H24	Red deer	*Cervus canadensis*	Highly medullated hair	~55	Wild
H25	Eurasian moose	*Alces alces*	Highly medullated hair	~45	Wild
H26	Yellow-throated marten	*Martes flavigula*	Lightly medullated hair[T]	~55	Wild
H27	Mountain hare	*Lepus timidus*	Lightly medullated hair[T]	~60	Wild
H28	Red fox	*Vulpes vulpes*	Lightly medullated hair[T]	~55	Wild
H29	Wolf	*Canis lupus*	Lightly medullated hair[T]	~45	Wild
H30	Leopard cat	*Prionailurus bengalensis*	Lightly medullated hair[T]	~55	Wild
H31	Leopard cat	*Prionailurus bengalensis*	Lightly medullated hair[T]	~55	Wild

H32	Wolf	*Canis lupus*	Lightly medullated hair	~1.5	Wild
H33	Siberian roe deer	*Capreolus pygargus*	Highly medullated hair	~3	Wild
H34	Amur tiger	*Panthera tigris altaica*	Lightly medullated hair	~6	Wild
H35	Amur tiger	*Panthera tigris altaica*	Lightly medullated hair	~6	Wild
H36	Amur tiger	*Panthera tigris altaica*	Lightly medullated hair	~6	Wild
H37	Amur tiger	*Panthera tigris altaica*	Lightly medullated hair	~6	Zoo
H38	Amur tiger	*Panthera tigris altaica*	Lightly medullated hair	~6	Zoo
H39	Amur tiger	*Panthera tigris altaica*	Lightly medullated hair	1	Zoo
H40	Amur tiger	*Panthera tigris altaica*	Lightly medullated hair	1	Zoo
H41	Amur tiger	*Panthera tigris altaica*	Lightly medullated hair	0.5	Zoo
H42	Amur tiger	*Panthera tigris altaica*	Lightly medullated hair	1	Zoo
H43	Amur tiger	*Panthera tigris altaica*	Lightly medullated hair	0.5	Zoo

Note: The symbol "~" indicates that the error of the preservation year is ± 5 years. The top corner marked T is the tanned sample.

Table S2 Information of sequencing data

Sample ID	Damage rate	R_m	A_L (bp)	Sample Type	Library ID
Skin					
S1	5.19%	26.23%	52.6	Dried raw skin	E1_M
S2	1.10%	88.04%	84.4		E2_M
S3	0.81%	92.83%	87.2		E3_M
S4	3.76%	66.68%	40.7		E4_M
S5	1.74%	49.15%	77.8		E5_M
S6	1.94%	51.05%	38.5		E6_M
S7	3.36%	73.07%	44.4		E7_M
S8	4.00%	69.51%	35.4	Tanned skin	E8_M
S9	2.73%	33.62%	63.4		E9_M
S10	1.78%	63.66%	39.1		E10_M
S11	1.10%	44.48%	32.0		E11_M
S12	2.48%	0.08%	24.9		E12_M
S13	2.75%	14.61%	32.7		E13_M
S14	0.87%	37.71%	91.0	Decaying skin	F1_M
S15	0.68%	38.70%	63.1		F2_M
Rootles hair shaft					
H1	1.55%	85.31%	64.2	Highly medullated hair	C1_H
H2/H33	2.35%	60.47%	34.7	Highly medullated hair	F2_H
H3/H23	4.08%	37.85%	44.0	Highly medullated hair	E5_H
H4	0.16%	79.59%	48.2	Highly medullated hair	A6_TL
H5/H24	1.73%	16.45%	39.8	Highly medullated hair	A5_TL

H6/H25	3.08%	34.62%	38.0	Highly medullated hair	E7_H
H7	1.77%	90.88%	63.1	Lightly medullated hair	A7_TL
H8/H40	1.10%	85.06%	71.9	Lightly medullated hair	G2H
H9/H19	2.55%	71.13%	47.6	Lightly medullated hair	A8_TL
H10/H20	2.39%	81.60%	41.7	Lightly medullated hair	A9_TL
H11/H32	3.02%	88.38%	46.0	Lightly medullated hair	C3_H
H12/H22	3.01%	74.06%	42.0	Lightly medullated hair	E4_H
H13	1.53%	91.90%	52.4	Lightly medullated hair	A4_TL
H14/H21	1.98%	83.27%	33.7	Lightly medullated hair	A10_TL
H15	2.61%	93.14%	47.3	Lightly medullated hair	C6_H
H16	2.48%	92.24%	54.6	Lightly medullated hair	C7_H
H17-A	2.38%	93.43%	51.6	Lightly medullated hair	D1_A
H17-B	3.09%	93.68%	43.4	Lightly medullated hair	D1_B
H17-C	2.17%	89.96%	41.7	Lightly medullated hair	D1_C
H18-A	3.56%	78.56%	52.2	Highly medullated hair	D2_A
H18-B	3.91%	79.70%	49.8	Highly medullated hair	D2_B
H18-C	4.74%	80.70%	43.1	Highly medullated hair	D2_C
H26	4.48%	53.92%	29.6	Lightly medullated hair[T]	A2_TL
H27	3.78%	53.13%	28.2	Lightly medullated hair[T]	A1_TL
H28	3.65%	53.02%	41.2	Lightly medullated hair[T]	A3_TL
H29	3.17%	39.51%	41.6	Lightly medullated hair[T]	E11_H
H30	1.93%	5.49%	31.0	Lightly medullated hair[T]	A11_TL
H31	2.35%	14.39%	32.1	Lightly medullated hair[T]	A12_TL
H34	1.02%	84.10%	78.5	Lightly medullated hair	G1F
H35	1.71%	78.99%	73.7	Lightly medullated hair	G2F
H36	1.37%	82.31%	73.9	Lightly medullated hair	G3F

H37	1.62%	82.73%	72.5	Lightly medullated hair	G4F
H38	1.70%	82.75%	72.5	Lightly medullated hair	G5F
H39	1.63%	84.29%	72.5	Lightly medullated hair	G1H
H41	1.13%	85.09%	72.0	Lightly medullated hair	G3H
H42	1.10%	82.31%	74.0	Lightly medullated hair	G4H
H43	1.08%	84.05%	78.4	Lightly medullated hair	G5H

Note: A_L is average read size, R_m is mapping rate. The two samples separated by "/" share the same dataset.

Table S3 Information for different sample types

Different types of hair shaft

Highly medullated hair			Lightly medullated hair		
ID	R_m	A_L (bp)	ID	R_m	A_L (bp)
H1	85.31%	64.2	H9	71.13%	47.6
H2	60.47%	34.7	H10	81.60%	41.7
H3	37.85%	44.0	H11	88.38%	46.0
H4	79.59%	48.2	H12	74.06%	42.0
H5	16.45%	39.8	H13	91.90%	52.4
H6	34.62%	38.0	H14	83.27%	33.7
H7	90.88%	63.1			
H8	85.06%	71.9			

Hair shaft digested in the digestive tract

Fresh hair			Hair collected from faeces		
ID	R_m	A_L (bp)	ID	R_m	A_L (bp)
H34	84.10%	78.5	H39	84.29%	72.5
H35	78.99%	73.7	H40	85.06%	71.9
H36	82.31%	73.9	H41	85.09%	72.0
H37	82.73%	72.5	H42	82.31%	74.0
H38	82.75%	72.5	H43	84.05%	78.4

Different tanning degree (hair shaft)

Non-tanned			Moderately tanned			Fully tanned		
ID	R_m	A_L (bp)	ID	R_m	A_L (bp)	ID	R_m	A_L (bp)
H19	71.13%	47.6	H26	53.92%	29.6	H30	5.49%	31.0
H20	81.60%	41.7	H27	53.13%	28.2	H31	14.39%	32.1
H21	83.27%	33.7	H28	53.02%	41.2			

H22	74.06%	42.0	H29	39.51%	41.6			
H23	37.85%	44.0						
H24	16.45%	39.8						
H25	34.62%	38.0						
Different tanning degree (skin)								
Non-tanned			Moderately tanned			Fully tanned		
ID	R_m	A_L (bp)	ID	R_m	A_L (bp)	ID	R_m	A_L (bp)
S1	26.23%	52.6	S8	69.51%	35.4	S12	0.08%	24.9
S2	88.04%	84.4	S9	33.62%	63.4	S13	14.61%	32.7
S3	92.83%	87.2	S10	63.66%	39.1			
S4	66.68%	40.7	S11	44.48%	32.0			
S5	49.15%	77.8						
S6	51.05%	38.5						
S7	73.07%	44.4						
Different sections of hair shaft								
Basal			Middle			Distal		
ID	R_m	A_L (bp)	ID	R_m	A_L (bp)	ID	R_m	A_L (bp)
H17-A	93.43%	51.6	H17-B	93.68%	43.4	H17-C	89.96%	41.7
H18-A	78.56%	52.2	H18-B	79.70%	49.8	H18-C	80.70%	43.1

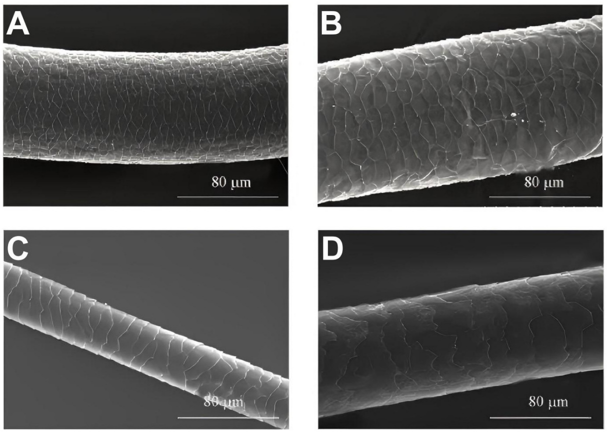

Figure S1. Scanning electron microscopy (SEM) images of different types of hair. (**A**) Red deer, (**B**) Siberian roe deer, representing coarse and highly medullated hair types; (**C)** Snow leopard, and (**D**) Leopard, representing fine and lightly medullated hair types. Scale bars: 80 µm.

DNA extraction and sequencing:

Genomic DNA was extracted from hair shafts (~10 mg) and skin tissue (~15 mg) using the Animal Genomic DNA Extraction Kit (Beijing Zhongkeleiming Technology Co. Ltd., Beijing, China; Cat. MB60909-100), incorporating a critical modification of Ca^{2+}-enhanced digestion (10 mM in buffer) at 56°C for 90 min. Library preparation was performed using the MGIEasy Universal DNA Library Prep Kit (MGI, Shenzhen, China; Cat. 1000006985), with procedural adaptation to accommodate naturally degraded DNA fragments (<100 bp). Specifically, DNA fragmentation was omitted, proceeding directly to end-repair and A-tailing steps. The libraries were sequenced on MGISEQ-2000RS high-throughput sequencer (MGI, Shenzhen, China) for 2×100 cycles. DNA quantification was performed using the Qubit™ 4.0 Fluorometer (Thermo Fisher Scientific, Waltham, MA, USA).

Significance test for differences：

Statistical analysis of R_m differences between lightly and highly medullated hair samples. (line 170)

(1) Normality Test (Shapiro-Wilk):

Highly medullated hair: W = 0.925, p-value = 0.5448 (normal distribution).

Lightly medullated hair: W = 0.907, p-value = 0.3345 (normal distribution).

(2) Homogeneity of Variance Test (Levene's Test):

F = 2.432, p = 0.1448 (homogeneity of variance assumed).

(3) t-Test (var.equal = TRUE):

t = -4.675, df = 12, p = 0.0005368 (significant difference).

Statistical analysis of R_{m0} differences between lightly and highly medullated hair samples. (line 171)

(1) Normality Test (Shapiro-Wilk)

Highly medullated hair: W = 0.72032, p-value = 0.01005 (non-normal).

Lightly medullated hair: W = 0.87464, p-value = 0.1672 (normal).

(2) Wilcoxon Test (Wilcoxon Rank-Sum Test)

W = 42, p-value = 0.01998 (statistically significant).

Statistical analysis of A_L differences between lightly and highly medullated hair samples. (line 171)

(1) Normality Test (Shapiro-Wilk)

Highly medullated hair: W = 0.96662, p-value = 0.869 (normal distribution).

Lightly medullated hair: W = 0.94939, p-value = 0.7051 (normal distribution).

(2) Homogeneity of Variance Test (Levene's Test)

F = 1.0829, p = 0.3186 (homogeneity of variance assumed).

(3) t-Test (var.equal = TRUE)

t = -1.2522, df = 12, p-value = 0.2344 (no significant difference).

Statistical analysis of R_m differences between lightly and highly medullated hair samples from untanned historical specimens. (line 213)

(1) Normality Test (Shapiro-Wilk)

Lightly medullated hair: W = 0.89856, p-value = 0.4239 (normal).

Highly medullated hair: W = 0.86024, p-value = 0.2682 (normal).

(2) Homogeneity of Variance Test (Levene's Test)

F = 0.2164, p = 0.6613 (homogeneity of variance assumed).

(3) t-Test (var.equal = TRUE)

t = 7.2997, df = 5, p-value = 0.0007554 (significant difference).

Statistical analysis of R_m differences between lightly medullated hair from untanned historical specimens and hair from tanned historical specimens. (line 213)

(1) Normality Test (Shapiro-Wilk)

Lightly medullated hair: W = 0.8987, p-value = 0.4246 (normal).

Tanned hair: W = 0.8073, p-value = 0.0683 (normal).

(2) Homogeneity of Variance Test (Levene's Test)

F = 2.2661, p = 0.1707 (homogeneity of variance assumed).

(3) t-Test (var.equal = TRUE)

t = 3.6501, df = 8, p-value = 0.006493 (significant difference).

Statistical analysis of R_m differences between highly medullated hair from untanned historical specimens and hair from tanned historical specimens. (line 215)

(1) Normality Test (Shapiro-Wilk)

Highly medullated hair: W = 0.86024, p-value = 0.2682 (normal).

Tanned hair: W = 0.8073, p-value = 0.0683 (normal).

(2) Homogeneity of Variance Test (Levene's Test)

F = 0.9484, p = 0.3626 (homogeneity of variance assumed).

(3) t-Test (var.equal = TRUE)

t = -0.51112, df = 7, p-value = 0.625 (no significant difference).

Statistical analysis of R_m differences between skin samples from untanned and tanned historical specimens. (line 221)

(1) Normality Test (Shapiro-Wilk)

Untanned skin: W = 0.95978, p-value = 0.8168

Tanned skin: W = 0.95346, p-value = 0.7682

(2) Homogeneity of Variance Test (Levene's Test)

F = 0.2016, p = 0.6621 (homogeneity of variance assumed).

(3) t-Test (var.equal = TRUE)

t = 1.8658, df = 11, p-value = 0.08894 (no significant difference).

Statistical analysis of A_L differences between lightly and highly medullated hair samples from untanned historical specimens. (line 229)

(1) Normality Test (Shapiro-Wilk)

Lightly medullated hair: W = 0.94109, p-value = 0.661 (normal).

Highly medullated hair: W = 0.95055, p-value = 0.5717 (normal).

(2) Homogeneity of Variance Test (Levene's Test)

F = 0.3461, p = 0.5819 (homogeneity of variance assumed).

(3) t-Test (var.equal = TRUE)
t = 0.17494, df = 5, p-value = 0.868 (no significant difference).

Statistical analysis of A_L differences between lightly medullated hair from untanned historical specimens and hair from tanned historical specimens. (line 229)

(1) Normality Test (Shapiro-Wilk)
Lightly medullated hair: W = 0.94109, p-value = 0.661 (normal).
Tanned hair: W = 0.81743, p-value = 0.08383 (normal).
(2) Homogeneity of Variance Test (Levene's Test)
F = 0.0847, p = 0.7784 (homogeneity of variance assumed).
(3) t-Test (var.equal = TRUE)
t = 1.9345, df = 8, p-value = 0.0891 (no significant difference).

Statistical analysis of A_L differences between highly medullated hair from untanned historical specimens and hair from tanned historical specimens. (line 229)

(1) Normality Test (Shapiro-Wilk)
Highly medullated hair: W = 0.95055, p-value = 0.5717 (normal).
Tanned hair: W = 0.81743, p-value = 0.08383 (normal).
(2) Homogeneity of Variance Test (Levene's Test)
F = 0.7183, p = 0.4247 (homogeneity of variance assumed).
(3) t-Test (var.equal = TRUE)
t = 1.7835, df = 7, p-value = 0.1177 (no significant difference).

Statistical analysis of A_L differences between skin samples from untanned and tanned historical specimens. (line 236)

(1) Normality Test (Shapiro-Wilk)
Untanned skin: W = 0.83897, p-value = 0.09719 (normal).
Tanned skin: W = 0.81937, p-value = 0.08715 (normal).
(2) Homogeneity of Variance Test (Levene's Test)
F = 2.2039, p = 0.1657 (homogeneity of variance assumed).
(3) t-Test (var.equal = TRUE)
t = 2.2517, df = 11, p-value = 0.04575 (significant difference).

Statistical analysis of R_m differences between fresh hair and hair collected from faeces. (line 257)

(1) Normality Test (Shapiro-Wilk)
Hair collected from faeces: W = 0.84505, p-value = 0.1794 (normal distribution).
Fresh hair: W = 0.85231, p-value = 0.2019 (normal distribution).
(2) Homogeneity of Variance Test (Levene's Test)
F = 0.2057, p = 0.6622 (homogeneity of variance assumed).
(3) t-Test (var.equal = TRUE)
t = -1.9995, df = 8, p-value = 0.08058 (no significant difference).

Statistical analysis of A_L differences between fresh hair and hair collected from faeces. (line 257)

(1) Normality Test (Shapiro-Wilk)

Hair collected from faeces: W = 0.76736, p-value = 0.04276 (non-normal).

Fresh hair: W = 0.77321, p-value = 0.04814 (non-normal).

(2) Wilcoxon Test (Wilcoxon Rank-Sum Test)

W = 16, p-value = 0.5476 (no statistically significant).

Statistical analysis of R_{dm} differences between hair and skin DNA. (line 276)

(1) Differences Normality Test (Shapiro-Wilk)

W = 0.93157, p-value = 0.4269 (normal distribution)

(2) Paired t-Test

t = 0.6566, df = 10, p-value = 0.5263 (no significant difference).

Statistical analysis of N_{db} differences between hair and skin DNA. (line 274)

(1) Differences Normality Test (Shapiro-Wilk)

W = 0.76021, p-value = 0.002826 (non-normal)

(2) Wilcoxon Test (Wilcoxon Signed-Rank Test)

V = 0, p-value = 0.05447 (no significant difference).
